# Validation of the Turkish Oral Health Impact Profile-17 for Assessing Oral Health–Related Quality of Life in Endodontic Patients

**DOI:** 10.3290/j.ohpd.c_2861

**Published:** 2026-11-11

**Authors:** Oğuz Burhan Çetinkaya, Betül Banu Gözübüyük, İrfan Yüksekkaya, Hasan Gündoğar

**Affiliations:** a Oğuz Burhan Çetinkaya Assistant Professor, Department of Endodontics, Faculty of Dentistry, Gaziantep University, 27500 Gaziantep, Türkiye; responsible for the conceptualisation, methodology, validation, formal analysis, investigation, and preparation of the original draft; b Betül Banu Gözübüyük Research Assistant, Department of Endodontics, Faculty of Dentistry, Gaziantep University, 27500 Gaziantep, Türkiye; responsible for the methodology, validation, investigation, data curation, and visualisation; c İrfan Yüksekkaya Research Assistant, Department of Endodontics, Faculty of Dentistry, Gaziantep University, 27500 Gaziantep, Türkiye; responsible for the validation, data curation, and the writing, review, and editing of the manuscript; d Hasan Gündoğar Associate Professor, Department of Periodontology, Faculty of Dentistry, Gaziantep University, 27500 Gaziantep, Türkiye; responsible for the conceptualisation and supervision of the study and the writing, review, and editing of the manuscript

**Keywords:** endodontics, oral health, quality of life, surveys and questionnaires, validation

## Abstract

**Objective:**

To evaluate the reliability and construct validity of the Turkish version of the Oral Health Impact Profile-17 (OHIP-17-TR) in patients receiving endodontic treatment.

**Materials and methods:**

This methodological, cross-sectional validation study included 229 adult patients undergoing or having recently completed endodontic treatment. Participants completed the OHIP-17-TR questionnaire. Internal consistency was assessed using Cronbach alpha and corrected item–total correlations. Construct validity was evaluated through exploratory factor analysis (EFA). Sampling adequacy was examined using the Kaiser–Meyer–Olkin (KMO) measure and Bartlett test of sphericity. Test–retest reliability was not assessed.

**Results:**

The OHIP-17-TR showed high internal consistency, with a Cronbach alpha of 0.894. Corrected item–total correlations ranged from 0.345 to 0.675. The KMO value was 0.882, and the Bartlett test was significant (*P* < 0.001), indicating suitability for factor analysis. Exploratory factor analysis identified a four-factor structure explaining 51.8% of the total variance, with all items demonstrating salient factor loadings. Subscale Cronbach alpha values ranged from 0.689 to 0.862.

**Conclusions:**

The OHIP-17-TR demonstrated good reliability and acceptable construct validity in this sample of endodontic patients. These findings support the use of OHIP-17-TR as a brief and practical patient-reported outcome measure for assessing oral health–related quality of life in endodontic research and clinical practice.

Oral health–related quality of life (OHRQoL) has become a key outcome in contemporary dentistry, complementing clinical indicators by capturing how oral conditions affect patients’ daily functioning, psychological well-being, and social interactions.^3,10
^ Patient-reported outcome measures (PROMs) are therefore increasingly recommended in both research and routine clinical practice to quantify the perceived burden of oral disease and the benefits of treatment from the patient’s perspective.^1,4
^


Among the most widely used PROMs in dentistry is the Oral Health Impact Profile (OHIP), which was developed to assess the frequency of negative impacts of oral conditions on quality of life.^22^ The original OHIP has several short forms, including OHIP-14 and OHIP-17, designed to reduce respondent burden while retaining strong psychometric performance.^12,23
^ Short-form instruments are particularly valuable in busy clinical settings, where rapid yet meaningful assessment is needed.^27^


Endodontic conditions and their treatment are closely linked to OHRQoL because symptoms such as pain, discomfort during chewing, sleep disturbance, and anxiety about dental procedures can markedly affect everyday life.^7,20
^ While endodontic therapy aims to relieve pain and restore function, patient-perceived outcomes may not always be fully reflected by clinical success criteria alone.^9,19
^ Consequently, a brief and reliable OHRQoL measure may provide additional insight for evaluating patient experiences and comparing treatment approaches in endodontic populations.

For a PROM to be used appropriately in a given language and cultural context, it must undergo translation and psychometric evaluation to ensure that it measures the intended construct reliably and validly.^3,11
^ Although Turkish versions of OHIP measures have been used in different dental contexts, evidence supporting the measurement properties of OHIP-17 in Turkish, particularly in an endodontic clinical population, remains limited.^2^ Establishing the reliability and construct validity of the Turkish version of the OHIP-17 (OHIP-17-TR) in endodontic patients would facilitate standardised outcome assessment and improve the comparability of findings across studies and clinical settings.^25^ The OHIP-17 was selected over the more widely used OHIP-14 because it includes three additional items addressing oral function and social disability that are particularly relevant to the symptom profile of endodontic patients.^7^ Specifically, these items capture dimensions of functional limitation and disability that may be more sensitive to the acute pain, chewing impairment, and social discomfort commonly reported by patients undergoing root canal treatment, thereby providing a more comprehensive assessment of the OHRQoL burden in this population compared with the 14-item version.^7,23
^


Therefore, the aim of the present study was to evaluate the reliability (internal consistency) and construct validity (factor structure) of the OHIP-17-TR in patients receiving endodontic care. The present authors hypothesised that the OHIP-17-TR would demonstrate high internal consistency and a coherent multidimensional structure suitable for assessing impacts on OHRQoL in this population.

## Materials and methods

### Study Design 

This study was designed as a methodological, cross-sectional validation study to evaluate the reliability and validity of the OHIP-17-TR in individuals undergoing endodontic treatment.^24^ The study protocol was approved by the Gaziantep University Non-Interventional Clinical Research Ethics Committee (approval number 2025/396, date 17 December 2025). Written informed consent was obtained from all participants. All procedures were conducted in accordance with the principles of the Declaration of Helsinki, and data confidentiality was strictly maintained.

### Study Setting and Participants

Participants were consecutively recruited from patients attending the Department of Endodontics, Faculty of Dentistry, Gaziantep University, between January and February 2026. A total of 229 participants were included in the final analysis.

The inclusion criteria were as follows:

age ≥18 years;undergoing or having recently completed endodontic treatment;ability to read and understand Turkish; provision of written informed consent.

The exclusion criteria were as follows:

cognitive/psychiatric conditions preventing reliable questionnaire completion;severe communication difficulties;acute systemic infection at the time of assessment;substantially incomplete questionnaires (e.g., missing responses in a considerable portion of OHIP items).

### OHIP-17-TR 

The OHIP-17 is a 17-item PROM assessing the negative impact of oral conditions on OHRQoL.^9^ Each item is rated on a 5-point Likert scale (0, never; 1, hardly ever; 2, occasionally; 3, fairly often; 4, very often). Total scores range from 0 to 68, with higher scores indicating poorer OHRQoL.

### Translation, Cultural Adaptation, and Content Validity

The OHIP-17-TR followed a standard forward–backward translation approach. Two independent bilingual translators produced forward translations into Turkish, which were then reconciled into a single Turkish version by the research team. Two different bilingual translators, blinded to the original, back-translated the reconciled Turkish version into English. The back-translated version was compared with the original instrument to ensure semantic and conceptual equivalence, and the necessary linguistic/cultural adjustments were made. An expert panel of clinicians/academics evaluated item relevance and clarity; the Content Validity Index (CVI) was computed at item and scale levels. The pre-final version was pilot tested in 25 individuals to confirm comprehensibility, and the final OHIP-17-TR was established.

### Data Collection Procedure

After eligibility screening and the provision of informed consent, participants completed a brief sociodemographic form (e.g., age, sex, education level, marital status) and the OHIP-17-TR questionnaire. Questionnaires were administered in a single session (chairside/clinic setting) with no time limit. Test–retest assessment was not performed; therefore, temporal stability (intraclass correlation coefficient [ICC]–based test–retest reliability) could not be evaluated.

### Statistical Analysis

Data were analysed using SPSS Statistics (version 22; IBM, Armonk, NY, USA). Statistical significance was set at *P* < 0.05. Continuous variables were summarised as mean ± standard deviation (SD) (or median [IQR], as appropriate), and categorical variables as frequencies and percentages. Internal consistency was assessed using a Cronbach alpha (α ≥ 0.70 was considered acceptable). Corrected item–total correlations and ‘alpha if item deleted’ values were also reported.

#### Construct validity

For exploratory factor analysis (EFA), sampling adequacy was evaluated using the Kaiser–Meyer–Olkin (KMO) measure and Bartlett test of sphericity. Factors were extracted using principal axis factoring with varimax rotation. A factor loading threshold ≥ 0.40 was applied. An exploratory rather than confirmatory analytic approach was specified a priori, because the dimensional behaviour of the OHIP-17 had not previously been examined in a Turkish-speaking endodontic population and because the conceptually formulated seven-domain structure of the original OHIP has frequently failed to replicate in empirical factor-analytic studies;^12,13
^ EFA therefore allowed the latent structure of the OHIP-17-TR to emerge from the data without imposing a potentially misspecified model. Factors were labelled post hoc according to the content of the items loading saliently on them.

#### Convergent/criterion validity 

No external criterion measure (e.g., global oral health rating or pain visual analogue scale [VAS] score) was systematically collected; therefore, criterion validity could not be assessed in the present study.

## Results

### Participant Characteristics

A total of 229 participants were included in the final analysis (Gaziantep University, January to February 2026). Missing item values were handled by mean imputation during preprocessing. The mean age was 32.39 ± 12.51 years (median 32 [interquartile range 20 to 42], range 18 to 70). The sample comprised 148 women (64.6%) and 81 mes (35.4%) (Table 1).

**Table 1 Table1:** Participant characteristics and OHIP-17-TR total score

Variable	Value	Additional information
Sample size	229	NA
Age (years)	32.39 ± 12.51	Median 32 [IQR 20–42], range 18–70
Sex	Female 148 (64.6%), male 81 (35.4%)	NA
OHIP-17-TR total score (0–68)	24.65 ± 12.92	Median 24 [IQR 15–35], range 0–60
Floor/ceiling effect (total score)	Floor (0): 1 (0.4%)	Ceiling (68): 0 (0.0%)
NA, not applicable.		

### OHIP-17-TR Descriptive Findings

The OHIP-17-TR total score ranged from 0 to 60, with a mean of 24.65 ± 12.92 and a median of 24 (interquartile range 15 to 35). A floor effect was minimal, with 0.4% of participants scoring 0 on the total score, and no participant reached the maximum possible score (ceiling effect 0%). Item mean scores ranged from 1.06 to 2.12, with the highest mean scores observed for OHIP4 (mean 2.12) and OHIP3 (mean 2.12) (Table 2).

**Table 2 Table2:** Item-level descriptive statistics and internal consistency indices (OHIP-17-TR)

Item	Mean ± SD	Median	IQR	Corrected item–total r	Alpha if deleted
OHIP1	1.09 ± 1.24	1	0–2	0.345	0.895
OHIP2	1.06 ± 1.19	1	0–2	0.435	0.892
OHIP3	2.12 ± 1.24	2	1–3	0.405	0.893
OHIP4	2.12 ± 1.29	2	1–3	0.469	0.891
OHIP5	1.82 ± 1.21	2	1–3	0.541	0.889
OHIP6	1.41 ± 1.32	1	0–2	0.459	0.892
OHIP7	1.57 ± 1.31	2	0–3	0.556	0.888
OHIP8	1.51 ± 1.27	1	0–2	0.630	0.886
OHIP9	1.47 ± 1.25	1	0–2	0.629	0.886
OHIP10	1.41 ± 1.22	1	0–2	0.675	0.884
OHIP11	1.69 ± 1.31	2	1–3	0.600	0.887
OHIP12	1.72 ± 1.34	2	1–3	0.548	0.889
OHIP13	1.18 ± 1.26	1	0–2	0.459	0.892
OHIP14	1.06 ± 1.21	1	0–2	0.623	0.886
OHIP15	1.21 ± 1.16	1	0–2	0.625	0.886
OHIP16	1.10 ± 1.17	1	0–2	0.653	0.885
OHIP17	1.12 ± 1.18	1	0–2	0.633	0.886


### Reliability (Internal Consistency)

The OHIP-17-TR demonstrated high internal consistency, with a Cronbach alpha of 0.894. Corrected item–total correlations ranged from 0.345 to 0.675, indicating acceptable to strong item discrimination. Cronbach alpha values if an item was deleted ranged from 0.884 to 0.895, suggesting that no single item disproportionately reduced the scale’s internal consistency (Table 2).

Based on the factor solution identified in EFA, subscale internal consistency coefficients ranged from 0.689 to 0.862.

### Construct Validity (EFA)

Sampling adequacy was supported by a KMO value of 0.882, and the Bartlett test of sphericity was significant (χ^2^(136) = 1677.86, *P* < 0.001), indicating suitability for factor analysis. EFA indicated a four-factor structure (eigenvalues > 1), explaining 51.8% of the total variance. After rotation, all items showed salient loadings (maximum absolute loading per item ≥ 0.435), with primary loadings ranging from 0.435 to 0.778, supporting a coherent latent structure of the OHIP-17-TR in this sample (Table 3). In terms of item content, Factor 1 comprised seven items reflecting broader psychosocial consequences of oral conditions (OHIP10–OHIP12 and OHIP14–OHIP17: difficulty relaxing, difficulty falling asleep, being awakened by oral problems, irritability, difficulty performing usual jobs, reduced life satisfaction, and inability to function) and was labelled psychosocial impact. Factor 2 comprised the two speech- and taste-related items (OHIP1 and OHIP2) and was labelled functional limitation. Factor 3 comprised three items (OHIP6, OHIP7, and OHIP13: having to alter the temperature of foods, feeling tense, and feeling embarrassed) and was labelled psychological discomfort and thermal sensitivity. Factor 4 comprised five items dominated by pain- and eating-related impacts (OHIP3–OHIP5, OHIP8, and OHIP9: painful aching, discomfort when eating, feeling self-conscious, unsatisfactory diet, and interrupted meals) and was labelled orofacial pain and eating-related disability (Tables 3 and 4). Several items, such as OHIP5, OHIP7, and OHIP8, additionally showed secondary loadings of 0.30 to 0.48 on neighbouring factors.

**Table 3 Table3:** Exploratory factor analysis (varimax rotation): absolute rotated loadings

Item	Factor 1	Factor 2	Factor 3	Factor 4
OHIP1		0.796		
OHIP2		0.806		
OHIP3				0.787
OHIP4				0.723
OHIP5	0.320		0.310	0.571
OHIP6			0.853	
OHIP7	0.406		0.641	
OHIP8			0.480	0.477
OHIP9			0.346	0.552
OHIP10	0.654			
OHIP11	0.741			0.331
OHIP12	0.721			0.354
OHIP13			0.747	
OHIP14	0.636		0.329	
OHIP15	0.761			
OHIP16	0.720			
OHIP17	0.758			
Empty cells represent loadings < 0.30 that were suppressed.				

**Table 4 Table4:** Subscale internal consistency (based on the EFA factor structure)

Subscale (EFA factor)	Items	Cronbach alpha
Factor 1 (psychosocial impact)	OHIP10, OHIP11, OHIP12, OHIP14, OHIP15, OHIP16, OHIP17	0.862
Factor 2 (functional limitation)	OHIP1, OHIP2	0.689
Factor 3 (psychological discomfort and thermal sensitivity)	OHIP6, OHIP7, OHIP13	0.737
Factor 4 (orofacial pain and eating-related disability)	OHIP3, OHIP4, OHIP5, OHIP8, OHIP9	0.781


Subscale internal consistency was evaluated according to the four-factor structure identified in the EFA. Cronbach alpha coefficients indicated good internal consistency for Factor 1 (α = 0.862) and acceptable internal consistency for Factors 3 and 4 (α = 0.737 and 0.781, respectively). Factor 2 showed comparatively lower reliability (α = 0.689), which is likely attributable to the subscale containing only two items, as the Cronbach alpha is sensitive to the number of items (Table 4).

## Discussion

The present study evaluated the psychometric properties of the Turkish version of the OHIP-17-TR in a sample of endodontic patients treated at Gaziantep University. Overall, the findings support that OHIP-17-TR is a reliable and structurally valid instrument for assessing OHRQoL in this population. The scale demonstrated high internal consistency (Cronbach α = 0.894), with acceptable-to-strong corrected item–total correlations, indicating that the items contribute meaningfully to the overall construct. Construct validity was further supported by EFA, which yielded a four-factor structure explaining 51.8% of the total variance with salient rotated loadings across items. Taken together, the results of the present study support the initial hypothesis and suggest that the OHIP-17-TR can be used as a practical PROM to quantify the perceived impact of endodontic conditions and treatment on daily functioning and well-being. From an endodontic perspective, these findings are clinically meaningful, as endodontic conditions are typically characterised by acute pain, functional limitation, and psychological discomfort. Therefore, incorporating PROMs such as the OHIP-17-TR may provide a more comprehensive assessment of treatment impact beyond traditional clinical indicators.

The four-factor structure observed in the EFA suggests that the OHIP-17-TR captures multiple dimensions of OHRQoL in endodontic patients. This finding is broadly consistent with previous validation studies reporting a multidimensional structure for short-form OHIP instruments, although the exact number of factors and item clustering may vary across languages, clinical contexts, and analytical decisions (e.g., extraction/rotation methods and sample characteristics).^12,13,23
^ Examination of item content indicates that the four factors are readily interpretable in relation to the conceptual model underlying the OHIP. Factor 2 reproduced the original functional limitation domain in its entirety (difficulty pronouncing words and worsened sense of taste). Factor 4 combined two of the three physical pain items (painful aching and discomfort when eating) with both physical disability items (unsatisfactory diet and interrupted meals), indicating that endodontic patients experience orofacial pain and its immediate consequences for eating as a single, undifferentiated dimension of impact, a plausible pattern in a population whose presenting complaints are dominated by pulpal and periapical pain; the self-consciousness item also loaded on this factor, suggesting that in this clinical context, self-conscious discomfort accompanies pain- and eating-related limitation. Factor 3 combined the remaining physical pain item, namely having to alter the temperature of foods, with the two remaining emotionally toned items (feeling tense and feeling embarrassed); thermal sensitivity is a hallmark and often anxiety-laden symptom of pulpal disease, and its clustering with tension and embarrassment may reflect the anticipatory distress and avoidance behaviour that this symptom provokes rather than item malfunction. Factor 1 merged the psychological disability items concerning relaxation and sleep with the social disability (irritability, difficulty performing usual jobs) and handicap (reduced life satisfaction, inability to function) domains, forming a broad psychosocial impact dimension. Thus, rather than contradicting the theoretical model, the four-factor solution largely represents combinations of adjacent original domains, with the seven conceptual dimensions collapsed into four empirically distinguishable clusters, with only two items (self-consciousness and thermal sensitivity) crossing conceptual boundaries in a clinically interpretable manner. The secondary loadings observed for a few items (e.g., OHIP5, OHIP7, and OHIP8) are likewise consistent with the partly overlapping, hierarchical nature of the OHRQoL construct rather than with item malfunction. This pattern also accords with the international OHIP literature. The seven-domain structure of the original instrument was formulated conceptually rather than derived empirically, and factor-analytic studies of OHIP versions in several languages have generally not reproduced it, instead supporting a smaller number of dimensions; in particular, a four-dimensional structure comprising oral function, orofacial pain, orofacial appearance, and psychosocial impact has been identified consistently across languages and underlies current recommendations for the use and scoring of OHIP versions.^12,13
^ The structure observed here aligns broadly with that model: Factor 1 corresponds closely to the psychosocial impact dimension, Factor 4 to orofacial pain and its function-related sequelae, and Factor 2 to oral function in the narrow sense of speech and taste, whereas Factor 3 blends a pain-related symptom (thermal sensitivity) with psychosocial distress. Accordingly, the divergence from the seven-domain description should not be interpreted as evidence against the cross-cultural equivalence of the OHIP-17-TR; it more likely reflects the strong intercorrelations among adjacent conceptual domains documented for the OHIP family of instruments.^12^ Nevertheless, because the original seven subscales were not empirically supported in this sample, the present authors recommend that OHIP-17-TR scores be interpreted primarily at the level of the total score, with the four EFA-derived subscales used descriptively until the structure has been confirmed in an independent sample. In our dataset, Factor 1 showed the strongest internal consistency (α = 0.862), whereas Factor 2 demonstrated comparatively lower reliability (α = 0.689), likely reflecting the limited number of items loading on that factor, an expected property of Cronbach alpha in brief subscales.^6,18
^ Differences between the factor pattern in the present study and those reported in other populations may be explained by the symptom profile and functional limitations typical of endodontic care–seeking patients, as well as cultural and linguistic nuances influencing how questionnaire items are interpreted.^3,11
^ Future studies should test the stability of this factor structure using confirmatory factor analysis in an independent sample and evaluate measurement invariance across key subgroups (e.g., sex and age categories).^5,26
^ It should be noted that the present findings are based on EFA, and the identified structure should be interpreted as preliminary. Confirmatory factor analysis (CFA) in independent samples is required to verify the stability and generalisability of this factor model.

From a reliability perspective, the overall OHIP-17-TR score performed strongly, supporting its use as a single summary measure in endodontic settings. Although the EFA-derived subscales showed acceptable-to-good internal consistency overall, the comparatively lower alpha observed for Factor 2 should be interpreted with caution because it contained only two items; Cronbach alpha is known to be sensitive to the number of items in a scale, and short subscales may yield lower coefficients even when inter-item consistency is adequate.^6,18
^ Thus, while the factor structure is informative for understanding the multidimensional nature of impacts captured by OHIP-17-TR, the present authors recommend prioritising the total score for routine clinical or research use unless future studies confirm stable subscale structures and reliability across independent samples.^25^ In addition, the absence of test–retest reliability assessment limits the ability to evaluate the temporal stability of the instrument. This aspect is particularly important in endodontic populations, where symptom perception may fluctuate over time. Future studies should assess stability using ICCs over a clinically stable interval.

Clinically, these findings suggest that OHIP-17-TR can serve as a practical PROM to capture the perceived burden of endodontic conditions and treatment on daily life. Endodontic complaints frequently involve pain, functional limitations, and psychosocial distress, and a relatively brief, 17-item instrument such as the OHIP-17 may help quantify these patient-centred outcomes in a time-efficient manner.^7^ In addition, incorporating OHIP-17-TR into routine assessment could facilitate communication about patient priorities, monitor symptom-related changes in quality of life changes, and support outcome comparisons across treatment protocols or clinical subgroups.^16^ However, the lack of clinical correlates such as pain intensity or treatment stage limits the interpretability of OHIP-17-TR scores in relation to disease severity. Integrating clinical parameters with patient-reported outcomes in future studies would enhance the clinical relevance of the findings.

Several strengths of the present study should be noted. Participants were consecutively recruited from a real-world clinical setting, and the sample size was adequate for factor analytic procedures, providing stable estimates for internal consistency and item performance. The scale showed consistently acceptable corrected item–total correlations and no evidence that any single item undermined reliability, supporting the coherence of the instrument in this population.^24^ Furthermore, the inclusion of a heterogeneous patient group may be considered both a strength and a limitation, as it reflects real-world clinical variability but may also introduce additional variability in responses.

This study also has limitations. First, test–retest reliability was not assessed, and thus temporal stability (ICC-based reliability) could not be evaluated. This represents a meaningful gap, as temporal stability is considered a core psychometric property for PROMs; future studies should assess it over an appropriate interval (e.g., 1 to 2 weeks) using intraclass correlation coefficients. Second, the study was conducted at a single centre over a limited time window, which may restrict generalisability to other regions, institutions, or different endodontic case mixes. Third, construct validity was examined using EFA; confirmatory testing of the identified structure (CFA) in an independent sample was not performed and is necessary to determine whether the factor model replicates.^5,26
^ Finally, missing values were handled through mean imputation; this approach may have marginally influenced descriptive statistics and factor-analytic results, and future work should consider more robust missing-data approaches, such as multiple imputation.^8,14
^ The absence of external validity testing (e.g., convergent validity with pain or global health measures) limits the ability to determine how well the instrument reflects clinically meaningful outcomes in endodontic patients.

Future studies should extend these findings by evaluating test–retest reliability over an appropriate interval (e.g., 1 to 2 weeks) using the ICC to establish temporal stability, a core requirement for validating PROMs, confirming the factor structure through CFA in an independent sample, and assessing measurement invariance across key subgroups (e.g., sex and age) to ensure comparability of scores.^5,26
^ In addition, responsiveness analyses (pre- and post-treatment) would be valuable to determine whether the OHIP-17-TR is sensitive to clinically meaningful changes following endodontic interventions.^17,21
^


## Conclusions

In conclusion, the present findings indicate that the OHIP-17-TR is a reliable and structurally valid instrument for assessing OHRLQoL in endodontic patients in this setting. Although further work is warranted to confirm temporal stability and replicate the factor structure, the OHIP-17-TR appears suitable for use as a brief, patient-centred outcome measure in endodontic research and clinical practice.

## References
